# Involvement of MicroRNAs in the Aging-Related Decline of CD28 Expression by Human T Cells

**DOI:** 10.3389/fimmu.2018.01400

**Published:** 2018-06-18

**Authors:** Nato Teteloshvili, Gerjan Dekkema, Annemieke M. Boots, Peter Heeringa, Pytrick Jellema, Debora de Jong, Martijn Terpstra, Elisabeth Brouwer, Graham Pawelec, Klaas Kok, Anke van den Berg, Joost Kluiver, Bart-Jan Kroesen

**Affiliations:** ^1^Department of Pathology and Medical Biology, University of Groningen, University Medical Center Groningen, Groningen, Netherlands; ^2^Rheumatology and Clinical Immunology, University of Groningen, University Medical Center Groningen, Groningen, Netherlands; ^3^Genetics, University of Groningen, University Medical Center Groningen, Groningen, Netherlands; ^4^Department of Internal Medicine II, Center for Medical Research, University of Tübingen, Tübingen, Germany; ^5^Cancer Solutions Program, Health Sciences North Research Institute, Sudbury, Ontario, Canada; ^6^Laboratory Medicine, University of Groningen, University Medical Center Groningen, Groningen, Netherlands

**Keywords:** T cell aging, senescence, CD28, IL-15, miRNA, miR-9, miR-23a~24-2

## Abstract

Loss of CD28 is a characteristic feature of T cell aging, but the underlying mechanisms of this loss are elusive. As differential expression of microRNAs (miRNAs) has been described between CD28+ and CD28− T cells, we hypothesized that altered miRNA expression contributes to the age-associated downregulation of CD28. To avoid the confounding effects of age-associated changes in the proportions of T cells at various differentiation stages *in vivo*, an experimental model system was used to study changes over time in the expression of miRNA associated with the loss of CD28 expression in monoclonal T cell populations at a lower or higher number of population doublings (PDs). This approach allows identification of age-associated miRNA expression changes in a longitudinal model. Results were validated in *ex vivo* samples. The cumulative number of PDs but not the age of the donor of the T cell clone was correlated with decreased expression of CD28. Principal component analysis of 252 expressed miRNAs showed clustering based on low and high PDs, irrespective of the age of the clone donor. Increased expression of miR-9-5p and miR-34a-5p was seen in clones at higher PDs, and miR-9-5p expression inversely correlated with CD28 expression in *ex vivo* sorted T-cells from healthy subjects. We then examined the involvement of miR-9-5p, miR-34a-5p, and the members of the miR-23a~24-2 cluster, in which all are predicted to bind to the 3′UTR of CD28, in the IL-15-induced loss of CD28 in T cells. Culture of fresh naive CD28+ T cells in the presence of IL-15 resulted in a gradual loss of CD28 expression, while the expression of miR-9-5p, miR-34a-5p, and members of the miR-23a~24-2 cluster increased. Binding of miR-9-5p, miR-34a-5p, miR-24-3p, and miR-27- 3p to the 3′UTR of CD28 was studied using luciferase reporter constructs. Functional binding to the 3′UTR was shown for miR-24-3p and miR-27a-3p. Our results indicate involvement of defined miRNAs in T cells in relation to specific characteristics of T cell aging, i.e., PD and CD28 expression.

## Introduction

Full activation of naive T cells requires binding of the T cell receptor (TCR) to antigens displayed by the major histocompatibility complex on antigen-presenting cells (APCs) (known as “signal one”) together with ligation of a T cell costimulatory receptor (“signal two”). The latter is archetypically mediated by CD28 on the T cell surface and CD80 or CD86 on the APC. This interaction results in complex signaling cascades which activate T cells, promote their differentiation, proliferation, and effector function, and mounts adaptive immune responses depending on clonally expanded, antigen-specific effector T cells and the subsequent generation of T cell memory (1). Costimulation *via* CD28 lowers the threshold for signaling *via* the TcR and triggers cytokine production. This allows T cells to respond to low abundance and low avidity antigens, and shapes T cell immunity by balancing the interplay between effector and regulatory T cells (1). The latter is especially important in focusing the immune response toward the pathogen, avoiding autoimmunity, and for downregulating the immune response upon pathogen clearance.

The composition and function of the T cell immune system in older people is characterized by lower proportions of naive T cells and higher proportions of memory T cells as a result of antigen exposure over the lifetime (2). Additionally, aging itself affects the characteristics of T cells within the naive and memory compartments and when these effects result in compromised functionality, these T cells can be designated “immunosenescent” (2–4). Developmentally programmed thymic involution at puberty results in an abrupt decline in the output of naive T cells, although residual thymic activity maintains the production of small numbers of such cells in most people in their 50s or 60’s. The diversity of the memory T cell pool increasingly reflects pathogen exposures over the lifetime, especially its focus on maintaining immune surveillance of latent viruses, e.g., CMV, EBV, and many other pathogens (5, 6). Overall, numbers and proportions of naive T cells decline, despite partial compensation by homeostatic proliferation of these cells in the periphery, which may also contribute to their aging phenotype (7, 8). Repeated clonal expansions of memory cells on rechallenge by specific pathogens, or continuous challenges by persistent pathogens, are thought to be instrumental for the overall differences observed between T cells in younger and older individuals (9, 10). At the cellular level, T cell aging is characterized by a multitude of changes in the expression of cell surface proteins. Most notably, a gradual decline in the expression of CD28 has been reported as a characteristic feature of aged T cells, mostly but not only due to the age-associated accumulation of late-stage memory cells which do not express this coreceptor (11, 12). The exact mechanisms involved in the aging-related decline of CD28 are unknown. Dissecting the differences in CD28 expression resulting from altered proportions of naive and memory T cells with age, and the intrinsic aging process within single T cell populations is challenging. To approach this, we have employed monoclonal T cells with increasing population doublings (PDs) in culture as a longitudinal aging model to identify regulation of CD28 expression, and attempted to validate some of these in *ex vivo* sorted T-cells from healthy subjects (13, 14) Here, we report the activity of microRNAs (miRNAs) in this context.

MicroRNAs are small noncoding RNA molecules that regulate protein expression by interfering with the process of messenger RNA (mRNA) translation or by inducing mRNA degradation. miRNAs are crucially involved in T cell development, differentiation, activation, and function (15, 16). In addition, recent evidence has implicated the involvement of miRNAs in several aspects of T cell aging (15–19). However, if and how miRNAs are involved in the regulated decline of CD28 expression is unknown. High expression of the three members of the miR-23a~24-2 cluster in CD8+CD28− T cells relative to CD8+CD28+ T cells has been reported (20). Increased expression of miR-24 in CD28− T cells was associated with an increased susceptibility to cell death, which was counterbalanced by IL-15 (20). IL-15 is a homeostatic cytokine that supports survival and proliferation of naive CD28+ T cells in the absence of continuous TCR stimulation (21). Downregulation of CD28 in response to homeostatic cytokines, such as IL-15, which interact with common γ-chain receptors, has been well documented (21–23). Here, we studied the involvement of miRNAs in clonal expansion and IL-15-regulated expression of CD28 by T cells. Using T cell clones derived from healthy young and elderly donors, we observed clustering of miRNAs primarily according to the number of PDs. In addition, IL-15 induced loss of CD28 coincided with upregulation of miRNAs that interact with the 3′UTR of CD28 mRNA.

## Materials and Methods

### Generation of T Cell Clones

T cell clones were generated from phytohemagglutinin-stimulated peripheral blood mononuclear cells (PBMC) by limiting dilution in the presence of IL-2 and pooled irradiated PBMC feeder cells as described previously (13, 24–26). In brief, cells to be cloned were plated at 0.45/well into 1 mm-diameter microplate wells containing 10^4^ 30 Gy-irradiated pooled PBMC from >20 random normal donors as feeder cells. Contents of positive wells were transferred after 1–2 weeks to 96-well 7 mm-diameter flat bottomed microtiter plate containing fresh medium and 10^5^ pooled PBMC feeder cells between day 7 and 11, and to 16 mm-diameter 24-well cluster plate with 2.5 × 10^5^ stimulators between day 12 and 16. Cultures were given fresh medium every 3 or 4 days and fresh feeder cells every 1–2 weeks thereafter. Clonal age is expressed in PD estimated by microscopic counting of the cells at each subculture and counting the number of doublings cumulatively undergone. Culture medium was the serum-free formulation X-Vivo 10 (BioWhittaker, Walkersville, MD, USA).

Three T cell clones from two healthy old and three T cell clones from one healthy young donor each at low and high PDs were selected for small RNA sequencing (*n* = 12 samples; three independent samples for each condition tested). Additional clones were used for the quantitative reverse-transcription-polymerase chain reaction (qRT-PCR) validation experiments (Table S1 in Supplementary Material). T-cell clones used were all CD4+. CD28 expression on T cell clones with low and high numbers of PDs was assessed by standard quantitative flow cytometry and expressed as median fluorescence intensity as reported previously (24, 25).

### Primary Lymphocyte Subsets

Peripheral blood mononuclear cells were freshly isolated by density gradient centrifugation using Lymphoprep (Axis-Shield, Oslo, Norway) according to the manufacturer’s protocol. Informed consent was obtained from all participants in accordance with the Declaration of Helsinki. The Medical Ethical Committee of the University Medical Center Groningen approved the study. For validation of differential miR-9-5p and miR-34a-5p expression in CD28+ versus CD28− T cells, CD3+CD28+ and CD3+CD28− cells were fluorescence-activated cell sorting (FACS) sorted from six healthy young (<30 years) and four healthy old (>60 years) subjects. For IL-15 culture experiments, CD3+CD8+CD45RO−CCR7+CD28+ T cells were FACS sorted from six healthy young (<30 years) subjects.

### FACS of Human Primary Lymphocyte Subsets and Analysis of Cell Surface Markers

The following monoclonal antibodies were used: anti-CD3-e450 (OKT3), anti-CD8a-APC-e780 (OKT8) (eBioscience, Vienna, Austria), anti-CD45RO-FITC (UCHL1), anti-CCR7-PE (3D12) (BD Bioscience, Breda, Netherlands), and anti-CD28 PeCy7 (CD28.2) (Biolegend, Uithoorn, The Netherlands). Cells were sorted using a MoFlo flow cytometry cell sorter (Backman Coulter, Woerden, The Netherlands).

Expression of cell surface markers on T cells was assessed using mAbs against human CD28-PE-CY7 (CD28.2) (Biolegend), CCR7-PE (3D12), and CD45RO-FITC (UCHL1) (BD Biosciences). Cells were analyzed using a BD LSR-II Flow Cytometer and the Diva software (BD Biosciences). Data analysis was done using the Kaluza Flow Analysis Software (1.2) (Beckman Coulter).

### T Cell Culture With Human Recombinant IL-15

Fluorescence-activated cell sorting-sorted CD3+CD8+CD28+CD45RO−CCR7+ (naive CD8+) T cells were suspended in RPMI medium (Lonza, Breda, The Netherlands) supplemented with 10 mg/ml gentamycin sulfate (Lonza) and 10% fetal calf serum (Thermo Scientific, Breda, The Netherlands) in a volume of 3 ml and seeded at a density of 1 × 10^6^/ml in T25 cm flasks. A final concentration of 50 ng/ml human recombinant IL-15 (Peprotech, London, UK) was added to the cell culture at day 0 and refreshed every 5th day. On day 5, 10, and 15 of culture, cells were harvested and stained for flow cytometry analysis and/or lysed for RNA isolation. To study CD28, CD45RO, and CCR7 expression on naïve CD8+ T cells after IL-15 stimulation, CD3+CD8+CD28+CD45RO−CCR7+ T-cells were sorted as described above and stained with 10 umol/ml eF670 proliferation dye (eBioscience, Vienna, Austria). After 5, 10, and 15 days of culture in the presence of IL-15 (50 ng/ml), cells were harvested, stained, and analyzed by flow cytometer.

### Culture of COS-7 Cells

COS-7 cells (African Green Monkey SV40-transformed kidney fibroblast cell line) were cultured in Dulbecco modified Eagle medium supplemented with 10% fetal bovine serum (Thermo Fisher Scientific, Breda, The Netherlands), 200 mM l-glutamine and 10 mg/ml gentamycin sulfate (Lonza, Breda, The Netherlands) at 37°C in 5% CO_2_.

### RNA Isolation

Total RNA was extracted using the miRNeasy Mini Kit (Qiagen, Venlo, The Netherlands) following the manufacturer’s instructions. Micro Bio-SpinTM chromatography columns, supplied with Bio-Gel P-6 polyacrylamide gel matrices, were applied to maximize purity of the RNA samples (Bio-Rad laboratories). The ExperionTM RNA stdSens and HighSens analysis kits (Life Science, Bio-Rad Laboratories B.V, Veenendal, The Netherlands) were used to determine the RNA quality indicator score. The RNA concentration was measured on a NanoDrop ND-1000 Spectrophotometer (NanoDrop Technologies, Wilmington, DE, USA).

### Small RNA Sequencing and Data Analysis

T cell clones with the biggest difference between low and high PDs were selected for small RNA-sequencing. Samples were barcoded and sequenced with Illumina HiSEQ 2000 flowcell (Illumina). The sequence reads were analyzed using the CLC BIO Genomic Work Bench Suite 4.5 (CLC BIO, Arhus, Denmark). Reads were mapped to the mature miRNAs using miRDeep2 (27). The number of mapped reads of each sample was normalized to 1 × 10^6^. Normalized data were imported to GeneSpring (v.11.5.1) for analysis. A total of 252 miRNAs were present in at least 3 out of 12 samples with a read count >10. Mann–Whitney *U* test was performed to identify significantly differentially expressed miRNAs. Genesis (Release 1.7.6) was used to generate heatmaps. Raw and processed data are available *via* the Gene Expresison Omninbus, accession #GSE106619.

### Quantitative RT-PCR

MicroRNA and gene expression levels were determined by qRT-PCR. cDNA synthesis for miRNAs was performed with Taqman miRNA Reverse Transcription kit using a multiplex reverse transcription approach with TaqMan microRNA Assays (Life Technologies, Carlsbad, CA, USA): for miR-9-5p (000583), miR-23a-3p (000399), miR-24-3p (000402), miR-27a-3p (000408), miR-31-5p (002279), miR-34a-5p (000426), and RNU44 (001094). RNU44 served as an endogenous control.

The qPCR reaction was performed using qPCR MasterMix Plus (Eurogentec, Liege, Belgium) and mean cycle threshold (Ct) values for all genes were quantified with the ViiA™ 7 software (Life Technologies). Relative expression levels were quantified using the 2−ΔC_t_ (ΔC_t_ = C_t_ gene − C_t_ reference gene) method.

### Cloning of 3′UTR in a Luciferase Reporter Construct, Transient Transfection, and Luciferase Reporter Assays

The CD28 3′UTR was cloned into the psiCHECK2 vector (Promega, Madison, WI, USA) in two fragments, as previously described (28). CD28 3′UTR-1 (nt 870-2279 of ENST00000324106.8) was amplified from genomic DNA using primers containing an Xhol (5') or Notl (3') restriction site, forward: 5′-GCTCCTGCACAGTGACTACA-3′, reverse 5′-ACCTTCTGCCTGACCACTTC-3′. CD28 3′UTR-1mut (same as above but with mutated miRNA binding sites), CD28 3′UTR-2 (nt 2534-4449), and CD28 3′UTR-2mut were ordered as minigenes (IDT, Leuven, Belgium). For both CD28 UTR-mut constructs mutations at position 2, 4, and 6 of the seed sequences were introduced at all potential miR-9-5p, -24-3p, -27-3p, and -34a-5p binding sites (based on sites indicated in Figure 5A). Sequences for the constructs are available on request. The inserts were sequence verified (BaseClear, Leiden, The Netherlands). COS-7 cells were transfected with 125 ng of the psiCHECK2 construct and either 50 nM hsa-miR-9-5p (PM10022), hsa-miR-24-3p (MC10737), hsa-miR-27a-3p (MC10939), hsa-miR-34a-5p (MC11030) mimics, or miRNA precursor negative control #1 (AM17110, ThermoFisher) using Saint-MIX (Synvolux products, Leiden, The Netherlands) following manufacturer’s instructions. Cells were lysed 48 h after transfection and Renilla and Firefly luciferase activity was assessed using Dual-Luciferase Reporter Assay System (Promega) according to manufacturer’s protocol. For each transfection, luciferase activity was measured in duplicate with the Luminoskan Ascent Microplate Luminometer (Thermo Scientific). The Renilla over Firefly (RL/FF) luciferase ratios were calculated and the RL/FF ratio of control precursor was set to one. All luciferase measurements were performed in at least three independent experiments.

### Statistical Analysis

For correlation analysis between miRNA or CD28 expression and PD the Spearman test was used. Paired samples as presented in Figures 2E,F were analyzed using the Wilcoxon signed-rank test and for Figures 3 and 4B,D (Figures S3B–D,H–J in Supplementary Material) and Figure 5 using the Friedman test with *post hoc* Dunnett’s multiple comparison test. Results obtained from luciferase assay were analyzed using the paired *T*-test. Statistical analyses were performed with GraphPad Prism version 7.0 (GraphPad Software, San Diego, CA, USA).

## Results

### PDs of T Cells Associates With Differential Expression of CD28 and miRNAs

**Figure 1:**
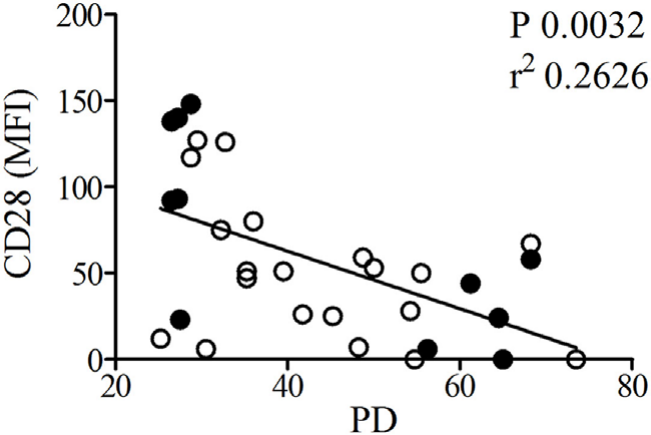
The population doublings (PDs) of T cell clones inversely correlate with CD28 expression. CD4+ T cell clones used for small RNA sequencing (filled symbols) with high and low PD and additional T cell clones (open symbols) were fluorescence-activated cell sorting analyzed for expression of CD28. Shown is the relation between CD28 expression based on mean fluorescence intensity (MFI) and PD of the T-cell clones. For one of the clones no MFI data are available.

For all 16 T cell clones, a passage at a lower number of PDs (≤40, median 29) and a passage at a high number of PDs (>40, median 56) was used (Table S1 in Supplementary Material). The Mean fluorescence intensity (MFI) of CD28 showed an inverse correlation with the number of PDs (Figure 1), which is in line with previous work (Figure 1) (14). No changes in CD28 expression were observed in the different age groups in which clones were grouped (data not shown). Small RNA sequencing was performed on the highest and lowest PD passage of 6 T cell clones. The T-cell clones used for this analysis were selected based on PD passages at the lowest and highest end of the PD spectrum.

**Figure 2:**
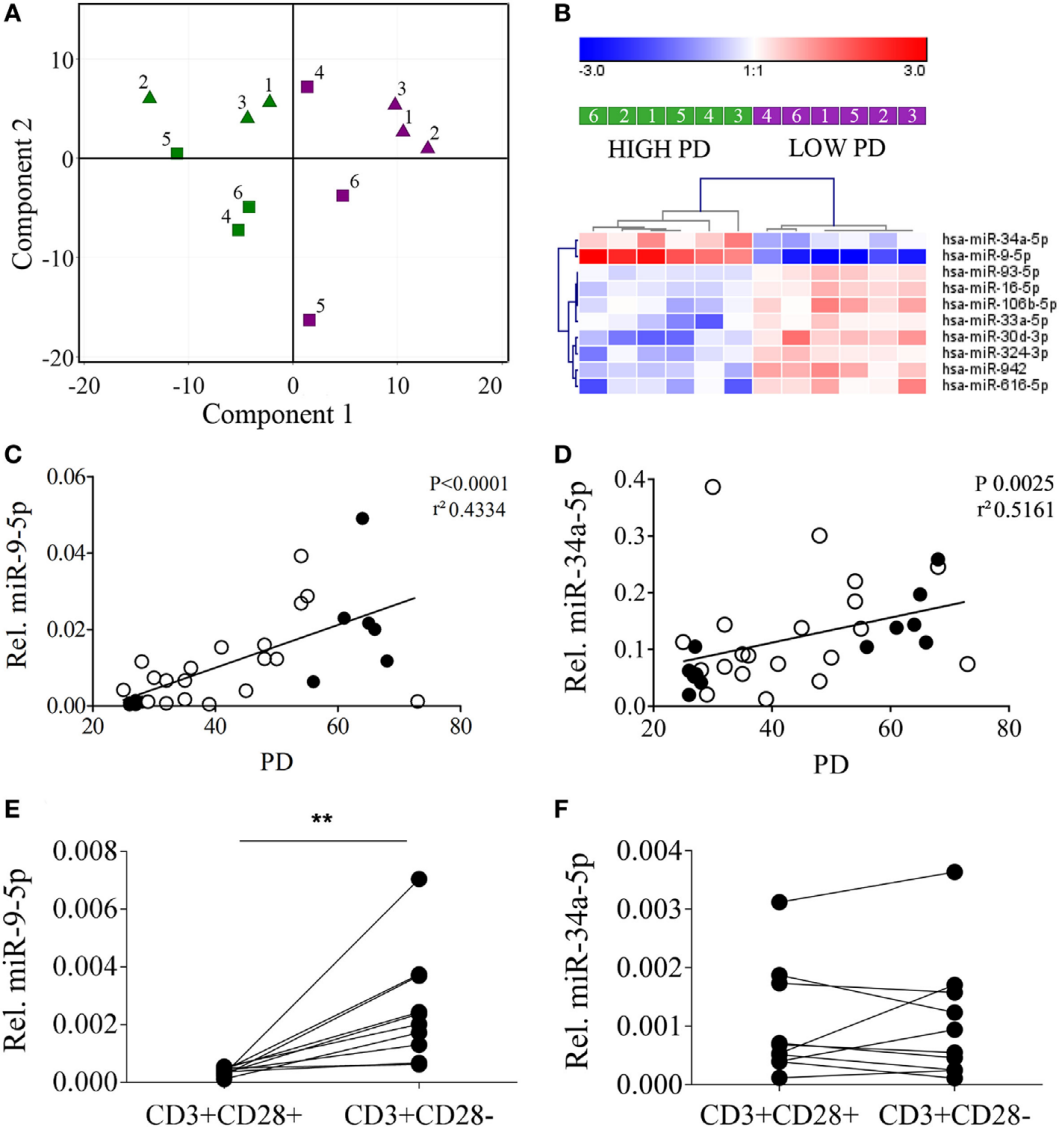
MicroRNAs (miRNA) expression analysis in CD4+ T cell clones reveals primarily clusters according to the proliferative history of the T cell clones and partly correlates with the expression of CD28. **(A)** Principal component analysis identifies low population doublings (PDs) (purple samples) versus high PDs (green samples) as the primary identifier of biological variation for miRNA expression. Triangles indicate young and squares old donors and numbers correspond to the T cell clone numbers indicated in Table S1 in Supplementary Material. **(B)** Hierarchical clustering of the T cell clones according to low and high PDs based on the 10 most significantly differentially expressed miRNAs. Correlation between **(C)** miR-9-5p and **(D)** miR-34a-5p and the PDs of the T cell clones used in the analysis. Filled symbols denote T cell clones used in the small RNA-sequencing samples and open symbols denote additional T cell clones used to validate data from small RNA-sequencing. Significant higher expression of **(E)** miR-9-5p but not **(F)** miR-34-5p in fluorescence-activated cell sorting-sorted CD3+CD28− T cells versus CD3+CD28+ T cells. Expression levels of the miRNAs relative to the expression of RNU44 is shown. Significance (**P ≤ 0.01) is depicted.

Principal component analysis of the 252 miRNAs detected in at least 3 of 12 samples revealed a perfect separation of the T cell clones in the first component based on PD (Figure 2A). No clustering was observed according to the age of the donor. Ten miRNAs were significantly differentially expressed between T cell clones with a low and a high number of PDs (Figure 2B). Five of these ten miRNAs were selected for validation based on having high expression levels and a more than 1.5-fold change in expression levels (see Table S2 in Supplementary Material). Validation was done by qRT-PCR on the 6 T cell clones that had been included for small RNA sequencing complemented with 10 additional T cell clones, also harvested at (intermediate) low and a high PD, giving a total of 32 samples (Table S1 in Supplementary Material). We observed a significant correlation for both miR-9-5p and miR-34a-5p levels with the number of PDs of the T cell clones (Figures 2C,D) and not for the other three miRNAs (data not shown).

### Independent Validation in Primary T Cell Subsets

To further validate the association between miR-9-5p and miR-34a-5p with aged T cells and CD28 loss, we sorted CD3+CD28+ and CD3+CD28− T cells from peripheral blood of six healthy young (<30 years) and four healthy old (>60 years) subjects. In line with the results obtained from the high and low PD T cell clones, qRT-PCR analysis revealed significantly higher levels of miR-9-5p in the CD3+CD28− T cell population as compared to the CD3+CD28+ T cells (Figure 2E). No significant differences were observed for miR-34a-5p (Figure 2F). Of note, lower relative expression levels of both miR-9-5p and miR-34a-5p were observed in the primary sorted T-cells, compared to the T-cell clones.

### Upregulation of miRNAs by IL-15 in Naïve CD8+ T Cells

Regulation of CD28 expression in CD8+ T cells has been described to occur downstream of IL-15. This prompted us to investigate whether IL-15 regulated the expression levels of miR-9-5p and miR-34a-5p. We also included miR-23a-3p, miR-24-3p, and miR-27a-3p all belonging to the miR-23a~24-2 cluster, in this analysis as differentially expression in CD28+ versus CD28− T cells has been previously described (20).

**Figure 3:**
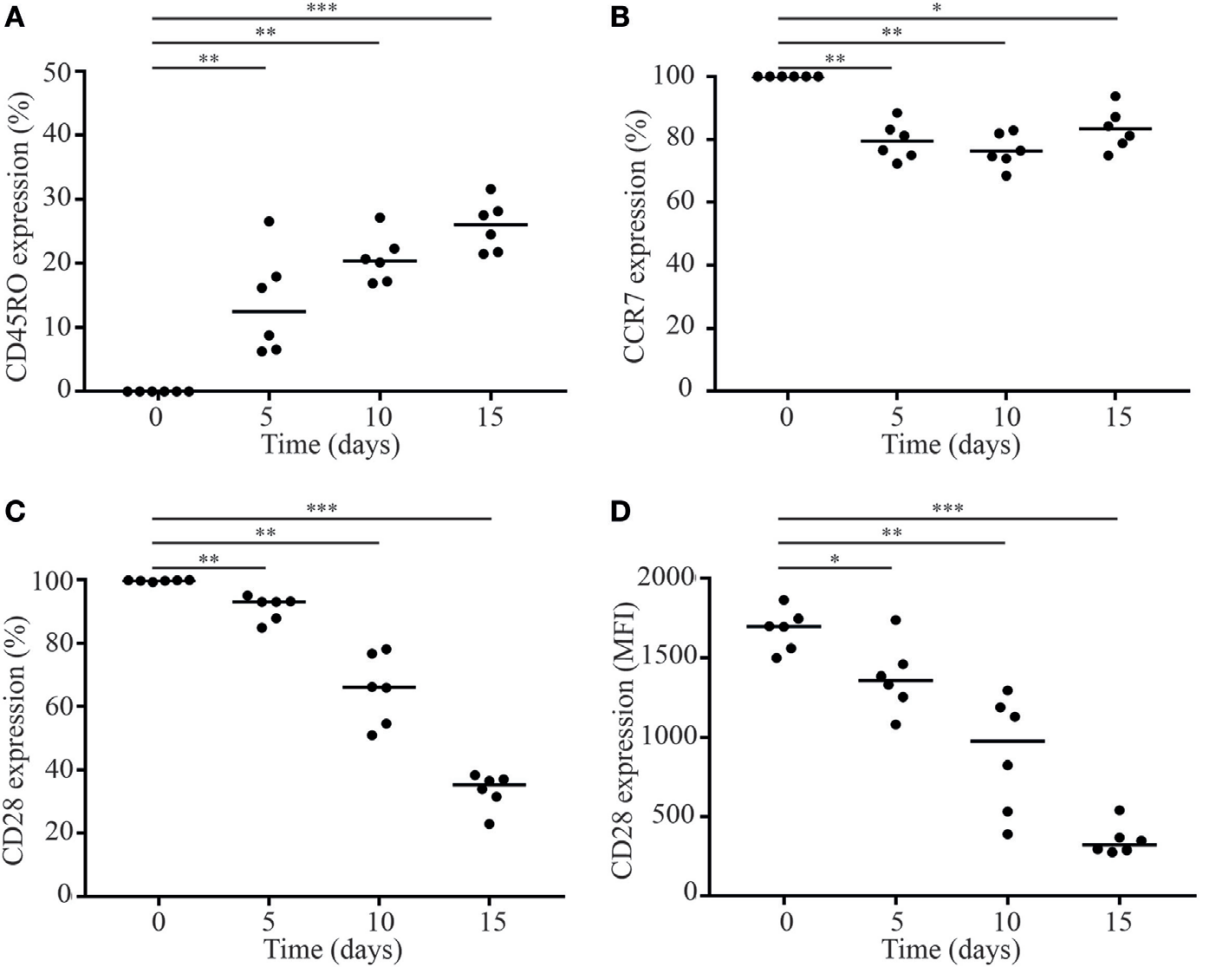
IL-15 induces loss of CD28 by naive CD8+ T cells. Fluorescence-activated cell sorting-sorted naive CD8+CD45RO−CCR7+CD28+ T cells were cultured in the presence of IL-15 (50 ng/ml). Directly after sorting and after 5, 10, and 15 days of culture, expression of **(A)** CD45RO, **(B)** CCR7, and **(C,D)** CD28 was assessed. MFI = median fluorescence intensity. Significance (*p < 0.05, **p < 0.01, ***p < 0.001) is depicted. *N* = 6.

To this end, we first sorted naive CD8+CD28+ T cells and cultured them for 15 days in the presence of IL-15 (Figure S1 in Supplementary Material). Culturing naive CD8+CD28+ T cells in the presence of IL-15 resulted in a shift to a memory phenotype as shown by a gain of CD45RO expression and a concomitant decrease in the expression of CCR7 (Figures 3A,B) and CD3 (data not shown). In line with the literature (21–23), we observed a significant downregulation of CD28 expression by naive T cells in response to IL-15. The percentage of CD28+ T cells decreased to 36% after 15 days culture in the presence of IL-15 (Figure 3C). Next to the decrease in the percentage of CD28+ T cells, also the expression of CD28 per cell was measured by the MFI decreased significantly (Figure 3D).

### Association Between miRNA Binding to the 3′UTR of CD28 and CD28 Expression

Next we studied whether IL-15-induced downregulation of CD28 was directly related to cell division. Analysis of sorted naïve T-cells stained with a proliferation dye revealed a progressive downregulation of CD28, both in terms of percentage positive cells and expression level, directly related to the number of cell divisions. Cells that did not divide retained CD28 expression. Similarly, cell division also correlated with acquiring a memory phenotype, as denoted by a loss of CCR7 and gain of CD45RO expression. These differences were most pronounced at day 15, but were also seen after 5 and 10 days stimulation with IL-15 (Figures S2 and S3 in Supplementary Material).

**Figure 4:**
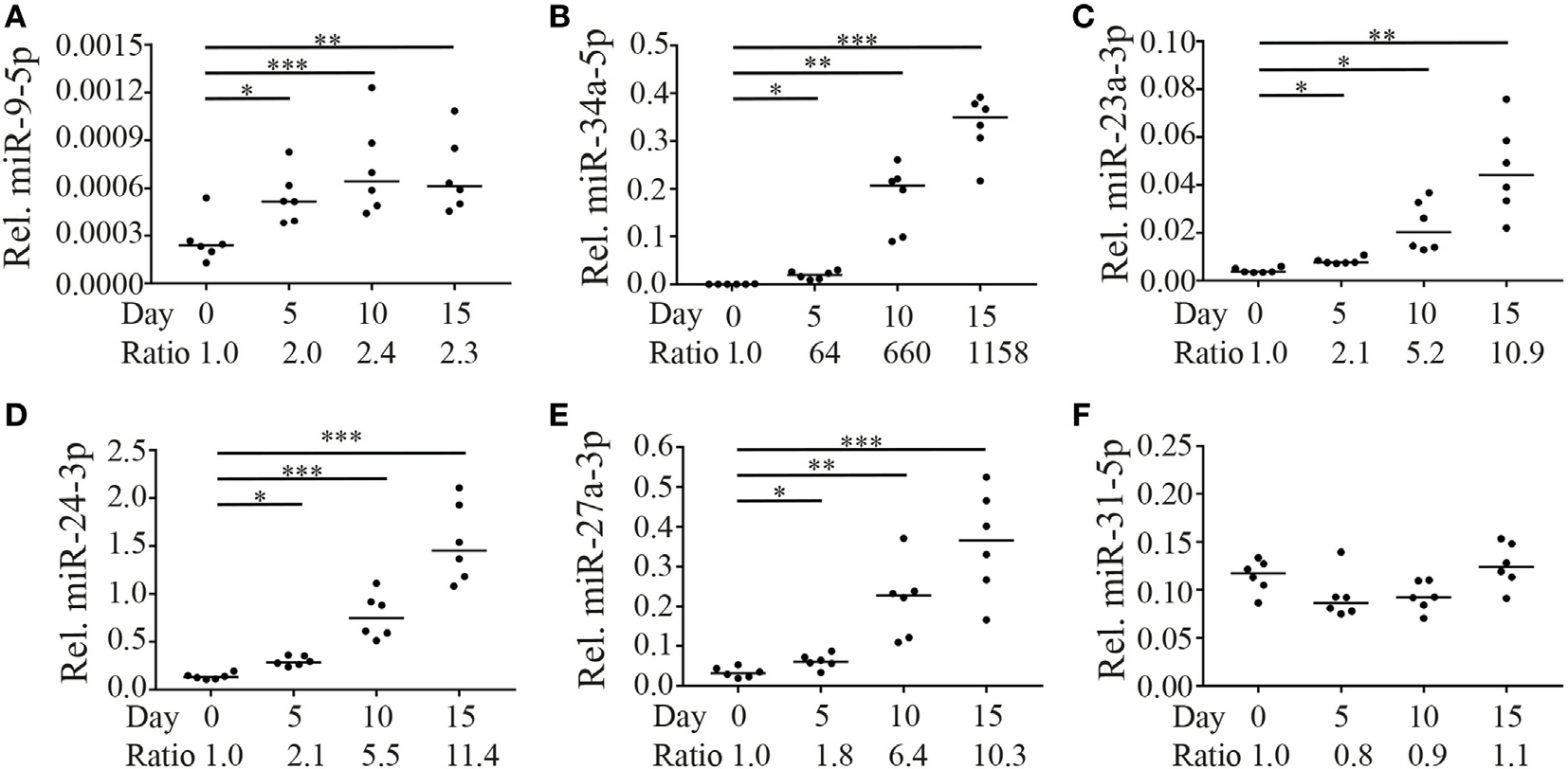
Expression of miR-9-5p, miR-34a-5p, and miR-23a~24-2 cluster in CD8+ naive T cells is regulated by IL-15. Fluorescence-activated cell sorting-sorted naive CD8+CD45RO−CCR7+CD28+ T cells were cultured in the presence of IL-15 (50 ng/ml). Directly after sorting and after 5, 10, and 15 days of culture, expression levels of **(A)** miR-9-5p, **(B)** miR-34a-5p, **(C)** miR-23a-3p, **(D)** miR-24-3p, **(E)** miR-27a-3p (all three part of the miR-23a~24-2 cluster), and **(F)** as a non-IL-15-responsive control, miR-31-5p were assessed by RT-qPCR. Expression levels of the microRNAs (miRNAs) relative to the expression of RNU44 are shown. Significance (*p < 0.05, **p < 0.01, ***p < 0.001) is depicted. *N* = 6.

Expression levels of the five selected miRNAs were assessed directly after sorting cells and after 5, 10, and 15 days of culture in the presence of IL-15. Expression of miR-9-5p, miR-34a-5p, miR-23a-3p, miR-24-3p, and miR-27a-3p increased over the 15 days of culture with similar kinetics for miR-34a-3p (Figure 4B) and the members of the miR-23a~24-2 family (Figures 4C–E). MiR-9-5p levels increased already at day 5, although to a lower extend (Figure 4A). Absolute expression levels significantly differed between the miRNAs with miR-24-3p having the highest and miR-9-5p the lowest expression levels. As a control, we tested the expression of a randomly selected unrelated miRNA (miR-31-5p), which did not significantly change as a result of stimulation with IL-15 (Figure 4F).

**Figure 5:**
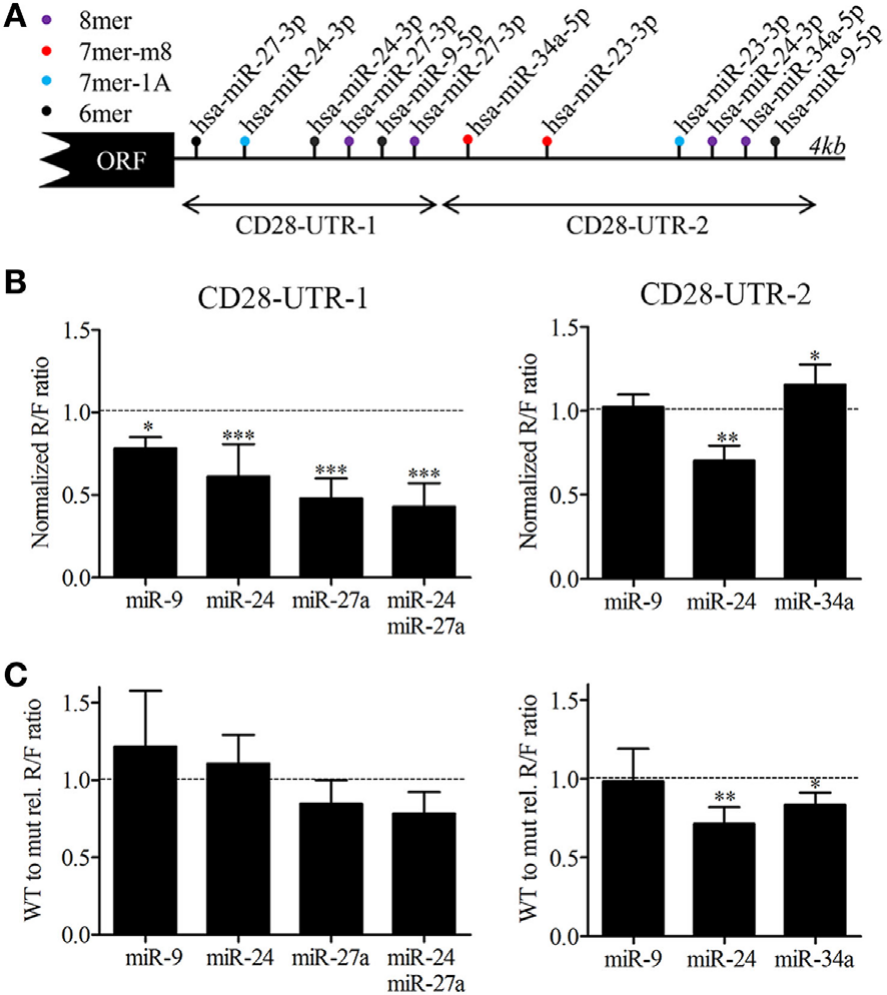
MicroRNAs (miRNAs) binding site analysis of the CD28 3′UTR. **(A)** Schematic overview of the predicted miRNA binding sites for miR-9-5p, miR-34a-5p, and members of the miR-23a~miR-24-2 cluster in the 3′UTR of CD28 (ENST00000324106.8). Binding sites were identified using the Targetscan prediction algorithm (release 7.1) in combination with a manual search for 6mers. The arrow indicates the two regions that were cloned in the luciferase reporter constructs. 8mer: exact match to positions 2–8 of the mature miRNA followed by an A, 7mer-m8: exact match to positions 2–8 of the mature miRNA, 7mer-A1: exact match to positions 2–7 of the mature miRNA followed by an A and 6mer: exact match to positions 2–7 of the mature miRNA. **(B)** Binding analysis of miR-9-5p, miR-24-3p, and miR-27a-3p to the proximal part of the CD28 3′UTR (CD28-UTR-1, left) and of miR-9-5p, miR-24-3p, and miR-34a-5p to the distal part (CD28-UTR-2 right). Cos-7 cells were transfected with psi-Check-2 construct harboring each fragment of the 3′UTR of CD28 **(A)** in combination with control precursor, or the miRNA(s) as indicated. The Renilla (R) over Firefly (F) luciferase ratio set to one for the control precursor transfected cells is shown. **(C)** The normalized R/F ratio of the wild-type CD28 UTR region relative (rel) to the normalized R/F ratio of the same region with mutated binding sites for the tested miRNAs. Significance (*p < 0.05, **p < 0.01, ***p < 0.001) is depicted. Each luciferase experiment was measured in duplicate and each experiment was performed at a minimum of three independent experiments.

Next, we identified putative miRNA binding sites in the 3′UTR of the CD28 mRNA. Using the Targetscan miRNA binding site prediction algorithm as well as manual searches for 6-, 7-, and 8-mer seed binding sites we identified two binding sites for miR-9-5p, miR-23a-3p, and miR-34a-5p and three binding sites for miR-24-3p and miR-27a-3p (Figure 5A). The presence of binding sites for these IL-15 responsive miRNAs in the 3′UTR of the CD28 transcript suggests a direct regulation. Two consecutive regions covering the 3′UTR of CD28 were cloned in a luciferase reporter construct for analysis to assess direct binding of miR-9-5p, miR-34a-5p, and the two most abundant members of the miR-23a~24-2 family (miR-24-3p and miR-27a-3p). In addition, we also generated CD28 3′UTR constructs in which the binding sites of the four miRNAs were mutated. COS-7 cells were transiently transfected with these constructs and specific miRNAs or control mimics. Luciferase assays using the wild-type (WT) CD28 3′UTR regions indicated binding of miR-9-5p, miR-24-3p, and miR-27a-3p to CD28-UTR-1 and of miR-24-3p to CD28-UTR-2 (Figures 5A,B). Comparison of WT to mutated constructs indicated that the relative R/F ratio of miR-27a-3p alone and in combination with miR-24-3p was reduced, albeit not significant (*p*-value = 0.11, *p*-value = 0.058, Figure 5C). For CD28-UTR-2 a reduced relative R/F ratio was observed for miR-24-3p and miR-34a-5p (*p*-value = 0.0062, *p*-value = 0.012, Figure 5C). Together these data suggest that miR-24-3p and miR-27a-3p are the most important miRNAs for direct regulation of CD28 expression.

## Discussion

Loss of CD28 is regarded as a hallmark of T cell aging (11, 14). To study T cell aging, various *in vitro* models have been applied, among which are T cell clones and IL-15 treatment of naive CD28+ T cells (21–24). We showed an inverse correlation between both miR-9-5p levels and miR-34a-5p, and CD28 expression in T cell clones with low and high PDs, in aged primary T cells and in IL-15 exposed naive T cells. In the latter model, we also showed IL-15 induced upregulation of the members of the miR-23a~24-2 cluster. Luciferase reporter assays showed targeting of the 3′UTR of the CD28 transcript by miR-24 and miR-27a, indicating miRNA dependent regulation of CD28 expression upon T cell aging.

The negative correlation between the number of PDs and the expression level of CD28 in T cell clones is consistent with their previously reported senescent phenotype (13, 14). We showed a clear PD-associated difference in miRNA expression levels in T cell clones, which was independent of the age of the T cell donor. These observations are consistent with previous findings showing an overall immune-biological similarity between T cell clones from centenarian and young adults (26, 29) and confirm that centenarian-derived T cell clones are, *per se*, not functionally compromised. We identified 10 miRNAs with a significant differential expression pattern in T cell clones with low and high number of PDs and confirmed an association with the number of PDs for miR-9-5p and miR-34a-5p. For miR-9-5p, we validated higher miR-9-5p levels in primary CD28− T cells as compared to CD28+ T cells isolated from both young and old subjects.

In a second model system used in this study, we assessed the kinetics of miR-9-5p and miR-34a expression in naive CD28+ T cells in response to IL-15. IL-15 expression increases with age and maintains survival of effector CD8+ T-cells. Increased expression of IL-15 in aged individuals has been implicated in the loss of CD28 expression by T-cells (6). In line with literature findings, culture of naïve CD8+ T cells with IL-15 induced a memory phenotype and concomitant gradual downregulation of CD28 in the proliferating cell fraction. We show that IL-15 mediated downregulation of CD28 by naïve CD8+CD28+ T cells is associated with a concomitant upregulation of miR-9-5p, miR-34a-5p, and the three members of the miR-23a~24-2 cluster. This confirms the previously reported high expression of the members of the miR-23a~24-2 cluster in CD28− T cells compared to CD28+ T cells (20). It remains to be established whether IL-15 induced miRNA expression involves other cytokines besides IL-15, such as TNF-a, which has also been implicated in the regulation of CD28 expression (30, 31).

Aging-related changes in expression of cell surface receptors are not restricted to CD28. Specifically, CD3γ regulated modulation of the TcR/CD3 complex has been reported in relation to aging (32). Downregulation of CD28 expression in our study was not seen as a result of stimulation by common γ-chain interacting cytokines *per se*, as culturing T cells in the presence of IL-4 did not induce downregulation of CD28 expression (data not shown). However, implication of the epigenome in the aging immune signature of memory CD8 T cells, involving silencing of the IL-7R gene and IL-7 signaling, has been described previously (33, 34).

As a mechanism to explain IL-15 induced loss of CD28 expression, we propose involvement of miRNAs targeting of the 3′UTR of the CD28 transcript. Indeed, several potential binding sites of miR-9-5p, miR-34a-5p, and miR-23a~24-2 cluster members are present in the 3′UTR of CD28. Using a luciferase reporter assay we confirmed binding of miR-24-3p and miR-27a-3p to the 3′UTR of the CD28 transcript, thus providing evidence for their functional involvement in the regulation of CD28 expression upon induction by IL-15.

We initiated our search for aging associated miRNAs with CD4+ T-cell clones. This allowed us to analyze both high and low PD samples of the same T cell clone. Because CD28 expression is regulated more profoundly in CD8+ T cells and previous studies have been conducted specifically using CD8+ T cells, we subsequently used sorted naïve CD8+ T cells to study IL-15 stimulation induced regulation of miRNAs and CD28 expression. We confirmed differential expression for miR-9-5p and miR-34a-5p. Between the various experimental settings, we noted considerable differences in the expression level of the miRNAs studied. Expression of miRNAs is cell type dependent and might, as such, explain differences observed between the various experimental settings.

We used a luciferase reporter system to verify functional binding of miR-9-5p miR-24-3p, miR-27a-3p and miR-34a-5p to the 3?UTR of CD28. Two approaches were followed, the first using WT 3′UTR constructs in combination with miRNA precursor overexpression and the second using 3′UTR constructs in which seed sequences of the miRNAs had been mutated. The combined analysis indicated that miR-24-3p and miR-27a-3p was the most effective in binding to the 3′UTR of CD28. It should be noted that results were obtained using COS-7 cells and it remains to be determined whether these results can be translated to T-cells. However, transducing T-cells directly with these constructs is not only technically complicated but also induces unpredictable activation-associated physiological changes.

The aging-related decline in CD28 expression is observed in human T cells but not in rodents. Notably, two of three miR-27a binding sites are missing in the 3′UTR of mouse CD28. On the other hand, the 3′UTR of mouse CD28 has one additional miR-24 binding site compared to the 3?UTR of human CD28. Such differences, but also other differences including higher or lower miRNA or target gene levels can explain differences in miRNA-mediated CD28 regulation between humans and rodents.

In conclusion, our results provide evidence for involvement of miRNAs in the process of replication-associated aging and IL-15-mediated regulation of CD28 expression. The data support a positive feedback loop in which IL-15 induces loss of CD28 and induction of miR-9 as well as the members of the miR-23a~24-2 cluster during T cell aging. The induced loss of CD28 and associated senescent phenotype of aged T cells may be stabilized or further enhanced by induction of the IL-15-induced miRNAs.

## Ethics Statement

Informed consent was obtained from all participants in accordance with the Declaration of Helsinki. The study was approved by the Medical Ethical Committee (METC) of the University Medical Center Groningen.

## Author Contributions

NT and GD performed experiments, prepared the data, and were involved in writing. PH supervised experiments and revised the manuscript. MT, PJ, and DJ performed experiments. EB, GP, and KK supervised the project and revised the manuscript. GP provided the T cell clones. AvdB, JK, AB, and B-JK initiated and supervised the project and the experiments and wrote the manuscript.

## Conflict of Interest Statement

The authors declare that the research was conducted in the absence of any commercial or financial relationships that could be construed as a potential conflict of interest.

## Supplementary Material

Figure S1 Sorting strategy of CD8+CD45RO−CCR7+CD28+T cells.

Figure S2 Loss of CD28 expression after 15 days culture with IL-15. Fluorescence-activated cell sorting-sorted naïve CD8+CD45RO−CCR7+CD28+ T cells were stained with proliferation dye to study CD28 expression after proliferation upon IL-15 stimulation (50 ng/ml). Proliferation of the naïve CD8 T cells was assessed after 15 days of culture. **(A)** Gate setting for the identification of the population doublings (PDs). Within the different PDs, **(B,E)** expression of CD28 per generation, **(C)** CD28 expression per cell per generation, **(D,F)** CD45RO, and **(F)** CCR7 expression was assessed. MFI = median fluorescence intensity. Significance (**p* < 0.05, ***p* < 0.01) is depicted. *N* = 3.

Figure S3 Loss of CD28 expression after 5 and 10 days culture with IL-15. Fluorescence-activated cell sorting-sorted naïve CD8+CD45RO−CCR7+CD28+ T cells were stained with proliferation dye to study CD28 expression after proliferation upon IL-15 stimulation (50 ng/ml). Proliferation of the naïve CD8 T cells was assessed after **(A–F)** 5 and **(G–L)** 10 days of culture. **(A,G)** Gate setting for the identification of the population doublings (PDs). Within the different PDs **(B,E,H,K)** expression of CD28 per generation, **(C,I)** CD28 expression per cell per generation, **(D,F,J,L)** CD45RO and **(F,L)** CCR7 expression was assessed. MFI = median fluorescence intensity. Significance (**p* < 0.05, ***p* < 0.01) is depicted. *N* = 3.
