# Supplementary figures and images for: Involvement of MicroRNAs in the Aging-Related Decline of CD28 Expression by Human T Cells
Source: Front Immunol. 2018 Jun 18;9:1400. doi: 10.3389/fimmu.2018.01400 (PMC6015875; doi:10.3389/fimmu.2018.01400)

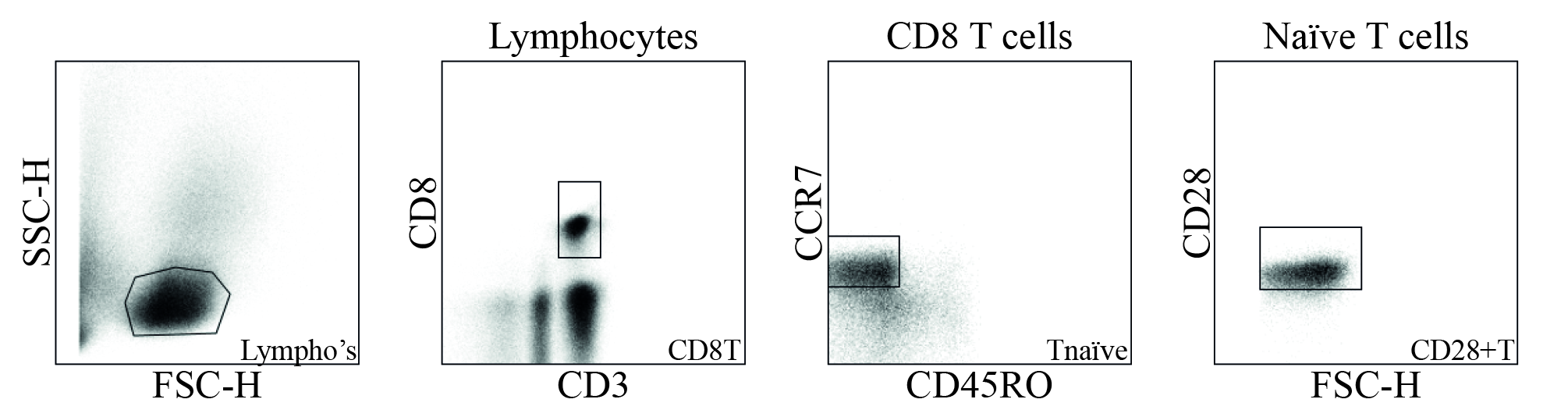

Supplement: Figure S1 — Sorting strategy of CD8+CD45RO−CCR7+CD28+T cells. [file image_1.tif]

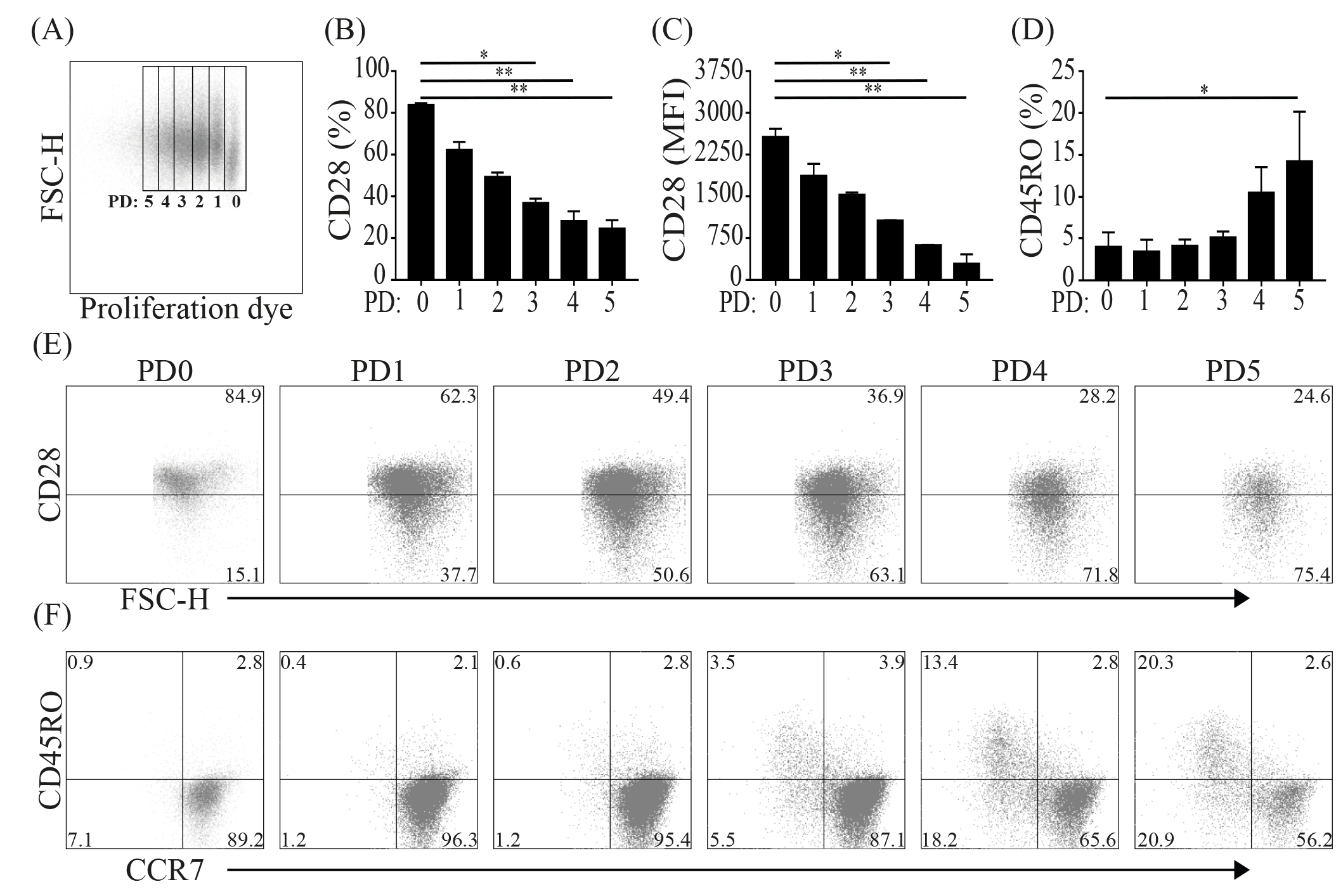

Supplement: Figure S2 — Loss of CD28 expression after 15 days culture with IL-15. Fluorescence-activated cell sorting-sorted naïve CD8+CD45RO−CCR7+CD28+ T cells were stained with proliferation dye to study CD28 expression after proliferation upon IL-15 stimulation (50 ng/ml). Proliferation of the naïve CD8 T cells was assessed after 15 days of culture. (A) Gate setting for the identification of the population doublings (PDs). Within the different PDs, (B,E) expression of CD28 per generation, (C) CD28 expression per cell per generation, (D,F) CD45RO, and (F) CCR7 expression was assessed. MFI = median fluorescence intensity. Significance (*p < 0.05, **p < 0.01) is depicted. N = 3. [file image_2.tif]

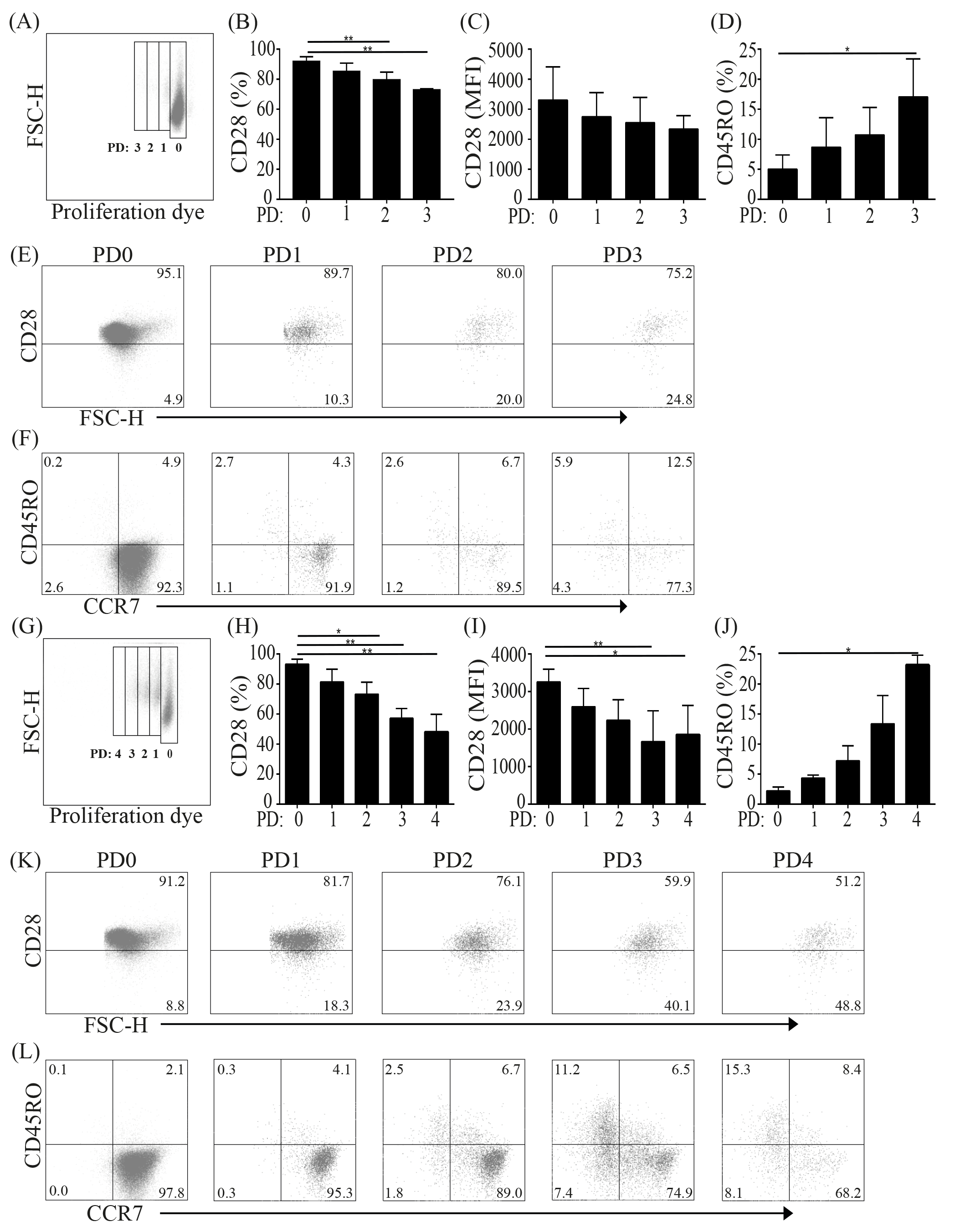

Supplement: Figure S3 — Loss of CD28 expression after 5 and 10 days culture with IL-15. Fluorescence-activated cell sorting-sorted naïve CD8+CD45RO−CCR7+CD28+ T cells were stained with proliferation dye to study CD28 expression after proliferation upon IL-15 stimulation (50 ng/ml). Proliferation of the naïve CD8 T cells was assessed after (A–F) 5 and (G–L) 10 days of culture. (A,G) Gate setting for the identification of the population doublings (PDs). Within the different PDs (B,E,H,K) expression of CD28 per generation, (C,I) CD28 expression per cell per generation, (D,F,J,L) CD45RO and (F,L) CCR7 expression was assessed. MFI = median fluorescence intensity. Significance (*p < 0.05, **p < 0.01) is depicted. N = 3. [file image_3.tif]
