# Supplementary material for: Involvement of MicroRNAs in the Aging-Related Decline of CD28 Expression by Human T Cells
Source: Front Immunol. 2018 Jun 18;9:1400. doi: 10.3389/fimmu.2018.01400 (PMC6015875; doi:10.3389/fimmu.2018.01400)
Supplement: Supplementary file 4 [file table_1.PDF]

Supplementary Table S1. T cell clone characteristics

Donors	*Age (yr)*	*Clone*	*Low PDs*		*High PDs*		*RNA-seq* *	*qPCR*
			PD	*MFI*	*PD*	*MFI*		
1	31	454-6	29	148	67	ND	+ (1)	+
		456-35	28	23	65	0	+ (2)	+
		456-42	27	138	56	6	+ (3)	+
		456-4	25	12	55	0	-	+
2	>85	433-26	27	92	61	44	+ (4)	+
		433-6	35	51	50	53	-	+
		433-9	33	126	49	59	-	+
		433-25	36	80	56	50	-	+
3	>85	434-29	29	117	42	26	-	+
4	100	461-15	27	93	65	24	+ (5)	+
		461-30	27	140	68	58	+ (6)	+
		461-17	40	51	68	67	-	+
		461-23	35	47	54	28	-	+
		461-33	32	75	74	0	-	+
5	100	460-31	31	6	48	7	-	+
		460-38	30	127	45	25	-	+

PD: population doubling; MFI: mean fluorescence intensity; ND: not determined;
*Numbers between brackets correspond to individual T cell clones shown in Figure 2A and B.
