# Supplementary material for: Involvement of MicroRNAs in the Aging-Related Decline of CD28 Expression by Human T Cells
Source: Front Immunol. 2018 Jun 18;9:1400. doi: 10.3389/fimmu.2018.01400 (PMC6015875; doi:10.3389/fimmu.2018.01400)
Supplement: Supplementary file 5 [file table_2.PDF]

Supplementary Table S2. Differentially expressed miRNAs at low and high number of PDs (P ≤0.05)

# ***miRNA***	***Regulation***	***Low PDs***	***High PDs***	***≥ 2 fold***
**hsa-miR-34a-5p**	**Up**	**262**	**486**	**1.9**
**hsa-miR-9-5p**	**Up**	**12**	**214**	**17.3**
**hsa-miR-16-5p**	**Down**	**16903**	**11475**	**1.5**
**hsa-miR-93-5p**	**Down**	**1151**	**831**	**1.4**
**hsa-miR-106b-5p**	**Down**	**263**	**152**	**1.7**
hsa-miR-33-5p	Down	30	20	1.5
hsa-miR-30d-3p	Down	36	14	2.5
hsa-miR-942	Down	20	10	1.9
hsa-miR-324-3p	Down	16	10	1.6
hsa-miR-616-5p	Down	11	6	2.0

Normalized read counts are shown (RPM); miRNAs selected for validation are highlighted in bold
